# Mitochondrial ROS, ER Stress, and Nrf2 Crosstalk in the Regulation of Mitochondrial Apoptosis Induced by Arsenite

**DOI:** 10.3390/antiox11051034

**Published:** 2022-05-23

**Authors:** Orazio Cantoni, Ester Zito, Andrea Guidarelli, Mara Fiorani, Pietro Ghezzi

**Affiliations:** 1 Department of Biomolecular Sciences, University of Urbino Carlo Bo, 61029 Urbino, Italy; ester.zito@uniurb.it (E.Z.); andrea.guidarelli@uniurb.it (A.G.); mara.fiorani@uniurb.it (M.F.); pietro.ghezzi@uniurb.it (P.G.); 2 Istituto di Ricerche Farmacologiche Mario Negri IRCCS, 20156 Milan, Italy

**Keywords:** arsenic, arsenite, mitochondrial ROS, endoplasmic reticulum stress, toxicity, Nrf2

## Abstract

Long-term ingestion of arsenicals, a heterogeneous group of toxic compounds, has been associated with a wide spectrum of human pathologies, which include various malignancies. Although their mechanism of toxicity remains largely unknown, it is generally believed that arsenicals mainly produce their effects via direct binding to protein thiols and ROS formation in different subcellular compartments. The generality of these mechanisms most probably accounts for the different effects mediated by different forms of the metalloid in a variety of cells and tissues. In order to learn more about the molecular mechanisms of cyto- and genotoxicity, there is a need to focus on specific arsenic compounds under tightly controlled conditions. This review focuses on the mechanisms regulating the mitochondrial formation of ROS after exposure to low concentrations of a specific arsenic compound, NaAsO_2_, and their crosstalk with the nuclear factor (erythroid-2 related) factor 2 antioxidant signaling and the endoplasmic reticulum stress response.

## 1. Introduction

Arsenicals are natural components of the Earth’s crust, normally present in traces in rocks, soil, water, air, plants, and animals. However, due to natural conditions or human activities [1], their levels are significantly increased in various regions, in particular in Bangladesh and West Bengal (India), in which human exposure to contaminated groundwater sources has been described by the World Health Organization (WHO) as “the largest mass poisoning of a population in history” [2]. Contamination of underground water occurs in other countries as well, including Chile, Argentina, Mexico, the USA, China, Taiwan, and Thailand [3].

Long-term exposure to arsenicals has been associated with an increased risk of skin, bladder, lung, kidney, and liver cancer [4] as well as with other non-cancerous diseases [4] and metabolism-related pathologies [5]. Furthermore, prenatal exposure to arsenicals impairs foetal brain development and cognition and increases the number of deaths in young adults [6].

Arsenic carcinogenesis has been associated with the induction of oxidative stress [7], genotoxicity [8], and epigenetic mechanisms [9].

Arsenic is a metalloid, sharing characteristics of both metals and non-metals. It exhibits a variety of oxidation states and can be found in the environment in different inorganic and organic forms in combination with other elements. The dominant forms are represented by trivalent and pentavalent arsenic, the former being remarkably more toxic than the latter [10]. Arsenic trioxide (As_2_O_3_), sodium arsenite (NaAsO_2_), and arsenic trichloride (AsCl_3_) are the most common inorganic trivalent compounds. Arsenical is metabolised via biomethylation, normally associated with reduced toxicity, with numerous reported exceptions [11].

NaAsO_2_, from now on defined as arsenite, avidly binds to -SH groups in proteins, thereby (possibly) altering their conformation, function, and interactions with other proteins. Consistently, various studies have shown that arsenite inactivates key cellular enzymes, especially those involved in the regulation of cellular energy pathways as well as DNA synthesis and repair [12].

Not surprisingly, arsenite also promotes the formation of reactive oxygen species (ROS) [11,13,14,15], commonly believed to account for both the cytotoxic and genotoxic effects of the metalloid [11,13,14,15,16,17], via yet poorly defined mechanisms, which however involve the mitochondrial respiratory chain [18,19,20] and the enzyme NADPH oxidase [21,22,23].

The notion that both the aforementioned mechanisms are Ca^2+^ dependent [24,25,26] implies that their recruitment is regulated by the relative concentrations of Ca^2+^ in specific sub-cellular microdomains, i.e., mitochondria for mitochondrial ROS formation or restricted cytosolic/membrane compartments for NADPH oxidase-derived ROS formation.

Thus, the effects of arsenic on Ca^2+^ homeostasis seem to be pivotal for the activation of a specific mechanism of ROS formation [27,28,29], a consideration bearing two important implications. The first one is that the endoplasmic reticulum (ER) represents a critical target of the metalloid [30,31,32,33,34,35], which is indeed considered a potent ER stressor [30,31,32,33,34,35]. The second implication is that arsenite potentially induces different Ca^2+^ responses and hence triggers different mechanisms of ROS formation in cell types characterised by a different functional organisation of their ER.

Deregulation of Ca^2+^ homeostasis also significantly impacts the triggering of specific toxicity mechanisms of the metalloid. An increased mitochondrial concentration of Ca^2+^ ([Ca^2+^]_m_), besides favouring the formation of mitochondrial superoxide (mitoO_2_^•−^), also promotes the mitochondrial permeability transition (MPT) and the MPT-dependent apoptosis [36,37,38]. MPT-independent mechanisms might on the other hand, mediate toxicity under conditions in which the metalloid elevates the cytosolic concentration of Ca^2+^ ([Ca^2+^]_c_) in the absence of significant increases in the [Ca^2+^]_m_ [39,40]. Furthermore, as indicated above, arsenite is an important ER stressor, and ER stress may critically crosstalk with, and contribute to, both MPT-dependent and -independent mechanisms of toxicity [41,42,43,44,45,46,47].

ROS formation is often associated with activation of nuclear factor (erythroid-2 related) factor 2 (Nrf2) antioxidant signaling [48,49]. Arsenite-derived ROS may, therefore, differentially activate this response when generated in different subcellular compartments. In addition, there are other potential mechanisms whereby arsenite may activate Nrf2 and promote survival.

In this review, the points raised above will be analysed and discussed. In particular, we will focus on the mechanism(s) whereby arsenite promotes mitochondrial Ca^2+^ accumulation and ROS formation, as well as on the crosstalk between these events and the Nrf2 and ER stress responses.

## 2. Arsenite Promotes the Mitochondrial Formation of Superoxide and Downstream Intra-Mitochondrial and Extra-Mitochondrial Effects

It is well known that mitochondria release significant amounts of ROS after stimulation mediated by various toxins [50,51]. Although multiple mechanisms contribute to this event [51,52], the respiratory chain by far represents the most efficient mechanism of mitoO_2_^•−^ formation [26,51,53]. Importantly, once released in the matrix, O_2_^•−^ is promptly converted by superoxide dismutase 2 (SOD2) to H_2_O_2_, which can now be degraded by glutathione (GSH) peroxidase, peroxiredoxins 3 and 5 or catalase, thereby preventing further reactions of the oxidant. However, the fraction of H_2_O_2_ escaping these interactions may eventually generate mitochondrial lesions through the formation of hydroxyl radicals, requiring the reaction of the oxidant with divalent iron (Fenton reaction). mitoO_2_^•−^, besides being a preferential substrate for SOD2-dependent conversion to H_2_O_2_, it can also interact with nitric oxide to generate the highly reactive peroxynitrite, thereby contributing to the formation of mitochondrial damage and dysfunction [18].

Importantly, mitoO_2_^•−^-derived H_2_O_2_ escaping metabolism by H_2_O_2_-detoxifying enzymes can also stimulate an array of signalling pathways [52,53] or generate deleterious effects on distal targets, such as various cytosolic proteins, genomic DNA, etc. [53]. These events are associated with the neutral and diffusible nature of the oxidant, which can easily cross the inner and outer mitochondrial membranes [26,51,53]. mitoO_2_^•−^ is also directly released in the intermembrane space [26], in which conversion to H_2_O_2_ is mediated by SOD1 [26], and its interaction with nitric oxide leads to the extra-mitochondrial formation of peroxynitrite [18].

The notion that arsenite under specific conditions generates mitoO_2_^•−^ is well documented [15,18,19,20], and the general idea is that this event is causally linked to the induction of mitochondrial dysfunction [18,19,41,42], and MPT-dependent apoptosis [41,42,43,44]. Likewise, mitoO_2_^•−^-derived H_2_O_2_ also mediates extra-mitochondrial effects, such as strand scission of genomic DNA [54], or the activation of the Nrf2 signalling pathway [55].

Our previous work has contributed to defining these events using U937 cells (which do not express nitric oxide synthase and are therefore unable to generate nitric oxide) exposed to low micromolar concentrations of arsenite. Under these conditions, the metalloid uniquely promoted the formation of mitoO_2_^•−^, i.e., without affecting ROS formation. The mitochondrial origin of the ROS induced by arsenite was established using various strategies, which include the use of specific fluorescent probes [56] and the inhibition of the activity of mitochondrial aconitase [54], an enzyme particularly sensitive to mitoO_2_^•−^ [57]. Moreover, ROS formation was suppressed by rotenone, a complex I inhibitor [58], or by the respiration-deficient phenotype [54], as well as by ascorbic acid supplementation [54], which is associated with the accumulation of millimolar mitochondrial concentrations of the vitamin [59]. Under the same conditions, we also observed suppression of early events, including DNA damage [54] and Nrf2 activation [55], as well as delayed mitochondrial dysfunction and apoptosis [60].

Thus, convincing experimental evidence documents the ability of arsenite to promote, under specific conditions, mitoO_2_^•−^ formation and a cascade of events leading to cyto- and genotoxicity.

## 3. ER-Mitochondria Crosstalk Regulates Arsenite-Induced mitoO_2_^•−^ Formation

Various agents have previously been reported to increase the rate of mitoO_2_^•−^ formation via Ca^2+^-dependent mechanisms [25,61,62], thereby implying that the onset of the ROS response is critically connected to other events causing an increased [Ca^2+^]_m_ [25,61]. The notion that arsenite affects Ca^2+^ homeostasis has been demonstrated in numerous studies [27,28,29,63]. This event, however, is not necessarily associated with the mitochondrial uptake of the cation. It is indeed well established that mitochondrial Ca^2+^ accumulation takes place via low-affinity transport mechanisms mediated by the mitochondrial Ca^2+^ uniporter (MCU) [62,64]. High Ca^2+^ concentrations are thus necessary for MCU-dependent transport, and these conditions are met at contact sites between the ER and mitochondria [65,66].

Recent studies from our laboratory have provided details of the effects of arsenite in the ER. Briefly, in keeping with the findings of others [28,63], we initially found that the inositol-1,4,5-triphosphate receptor (IP_3_R) is a primary target of the metalloid [67]. However, we also noticed that the resulting release of Ca^2+^, while limited and mediated by a saturable mechanism, was nevertheless critical to promoting a further release of the cation from the ryanodine receptor (RyR) [67]. Most importantly, the fraction of Ca^2+^ accumulated by the mitochondria was entirely derived from the RyR [54], thereby implying the relevance of this Ca^2+^ channel in processes associated with arsenite-induced mitochondrial Ca^2+^ accumulation. A cause–effect relationship between the increased [Ca^2+^]_m_ and the mitoO_2_^•−^ formation was next established by showing remarkable similarities in the time-dependence of these events [68], as well as the suppression of mitoO_2_^•−^ formation mediated by treatments preventing Ca^2+^ mobilisation from the IP_3_R or RyR or the transport of the cation in mitochondria [56]. Consistently, arsenite failed to increase the [Ca^2+^]_m_ and to promote the formation of mitoO_2_^−^^.^ in cell types, which do not express the RyR, such as HeLa cells, differentiated U937 cells, or undifferentiated C2C12 cells [56].

Thus, arsenite causes mitoO_2_^•−^ formation only in cell types, such as undifferentiated U937 cells or differentiated C2C12 cells, concomitantly expressing the IP_3_R and the RyR, with the latter channel in a close spatial and functional connection with the mitochondria [56].

We recently determined the mechanism regulating the crosstalk between the IP_3_R and the RyR, apparently based on the activation/expression of ER oxidoreductin 1α (ERO1α) [69]. More specifically, we found that the fraction of Ca^2+^ released by the IP_3_R (and RyR) was critical to activating ERO1α and that ERO1α was critical to promoting Ca^2+^ release from the RyR in microdomains sensed by the mitochondria [69]. Given the importance of the increased [Ca^2+^]_m_ in events associated with the mitoO_2_^•−^ formation, it appears clear that the above positive feedback amplification cycle is pivotal for the regulation of this response in cells exposed to arsenite.

However, the increased [Ca^2+^]_m_ represents a condition necessary but not sufficient to promote the mitoO_2_^•−^ formation, which in fact requires additional effects in the mitochondrial respiratory chain [68]. It was therefore interesting to observe that the concentration and time of exposure requirements for the effects of arsenite in the mitochondrial respiratory chain were significantly lower than those necessary for the triggering of the Ca^2+^ responses described above [68]. Remarkably, a 10 min exposure to RyR or IP_3_-releasing agonists, which rapidly increase the [Ca^2+^]_m_ [68], was sufficient to induce maximal mitoO_2_^•−^ emission in the presence of very low concentrations of arsenite. Thus, the effects of arsenite in the mitochondrial respiratory chain are also necessary but not sufficient and, most importantly, present much lower concentration and time of exposure requirements in comparison with Ca^2+^ mobilisation and mitochondrial accumulation.

Based on the observed Ca^2+^-dependence and sensitivity to rotenone, our findings are in keeping with the possibility that arsenite promotes its effects in the mitochondrial respiratory chain via a mechanism involving inhibition of complex III, a notion that should be experimentally established. Other studies have reported that arsenite effects follow a nonlinear dose-response in which low concentrations promote increased expression and activity of complex I and, under the same conditions, elicit cytoprotective signalling [19]. The effects mediated by the high concentrations were instead associated with reduced activity of the electron transport chain. These results, while interesting, are difficult to compare with those obtained in our studies since specific questions related to the concomitant changes in Ca^2+^ homeostasis were not addressed. In particular, the lack of complex III inhibition could depend on the lack of mitochondrial Ca^2+^ accumulation.

**Figure 1:**
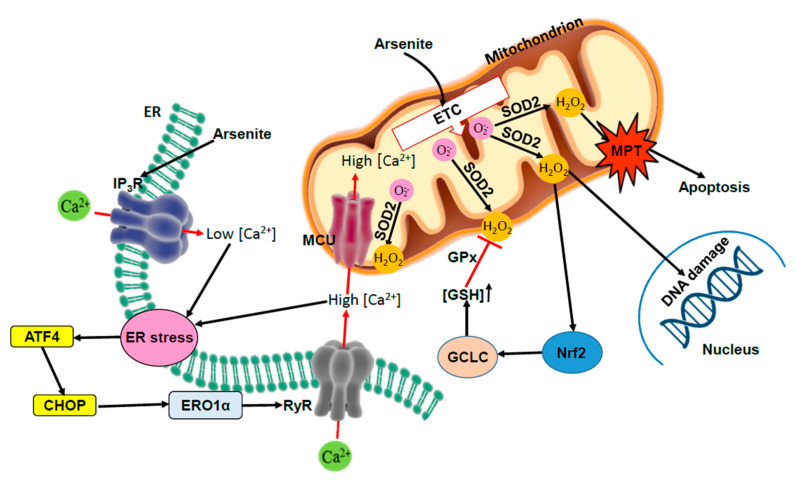
Crosstalk between arsenite-induced mitochondrial ROS, ER stress, and Nrf2. Arsenite promotes mitoO_2_^•−^ formation via a mechanism requiring interactions with the mitochondrial respiratory chain and an accumulation of Ca^2+^ in these organelles. The metalloid initially stimulates Ca^2+^ release from the IP_3_R, which, although not directly taken up by the mitochondria, nevertheless contributes to this event via RyR stimulation. Indeed, due to the close apposition with the mitochondria, only the fraction of Ca^2+^ the RyR can be taken up by the mitochondria. Cells uniquely expressing the IP_3_R, in which these channels are in close contact with the mitochondria, failed to generate mitoO_2_^•−^ in response to arsenite. RyR activation was regulated by ERO1α and the resulting mitochondrial accumulation of Ca^2+^ was critical for the formation of mitoO_2_^•^. In this perspective, while the ER stress response appears upstream to mitoO_2_^•−^ formation, it is nevertheless reasonable to predict that persistent mitoO_2_^•−^-derived H_2_O_2_ promotes mitochondrial dysfunction and toxicity. The early ER stress response was also critically connected through mitoO_2_^•^ formation with the triggering of the Nrf2 cytoprotective signaling, which indeed significantly mitigated and delayed the onset of MPT-dependent apoptosis.

We, therefore, conclude that the process of mitoO_2_^•−^ formation requires two separate effects induced by arsenite. The first one is rapidly induced by low concentrations of the metalloid at the level of the mitochondrial respiratory chain, whereas the second, much slower and requiring greater arsenite concentrations, is targeted on the ER and associated with Ca^2+^ mobilisation from the IP_3_R, the recruitment of the RyR regulated by ERO1α and the mitochondrial accumulation of the cation (Figure 1).

## 4. Effect of Arsenic on Nrf2 and Its Target Genes

Cells respond to potentially toxic compounds with several adaptive mechanisms. One such mechanism is represented by the induction of phase II drug-metabolizing enzymes in response to electrophiles via the electrophile response element, which is present in several genes involved in the detoxification of xenobiotics [70]. This definition was then broadened to that of antioxidant response element (ARE) [71]. Several transcription factors bind ARE sequences, particularly Nrf2 [71,72], which is considered a master regulator of the transcription of several genes coding for enzymes with antioxidant functions.

Nrf2-regulates antioxidant defence genes, including GSH biosynthetic enzymes, antioxidant enzymes, and the GSH-regenerating enzyme, glutathione reductase (GR) [73,74]. Other Nrf2 target genes are important in maintaining protein thiols in the reduced state and include thioredoxin (Trx), Trx reductase, and sulfiredoxin, as well as genes involved in energetic metabolism, iron metabolism, survival, proliferation, autophagy, proteasomal degradation, DNA repair, and mitochondrial physiology [72,75]. In addition, Nrf2 activation is associated with the induction of mitochondrial antioxidant enzymes such as Trx reductase-2, peroxiredoxins 3 and 5, GSH peroxidase, and SOD2, highlighting a role for Nrf2 in the control of mitochondrial redox homeostasis [76]. Nrf2 also regulates mitochondrial biogenesis by influencing the expression levels of coactivators and critical transcription factors [74,77,78].

In the absence of oxidants/electrophiles, Nrf2 associates with Kelch ECH-associated protein 1 (KEAP1), which, in association with cullin 3, targets Nrf2 for ubiquitinylation and proteasomal degradation. In the canonical pathway of Nrf2 activation, electrophiles and ROS react with KEAP1 cysteines, affecting their conformation and thus impeding Nrf2 ubiquitylation [79]. As a result, Nrf2 translocates into the nucleus and activates the transcription of genes containing the ARE. Once the levels of ROS get low, KEAP1 turns again Nrf2 signalling off [80].

The finding of the redox sensitivity of Nrf2 has emphasised its importance in the adaptation to ROS rather than just xenobiotics. Consistent with this, experiments with Nrf2 knockout mice have shown its importance in protecting from pulmonary oxygen toxicity through the induction of ROS-detoxifying enzymes [81] and in hyperoxia-induced retinopathy of prematurity [82]. Nrf2 is also activated in post-ischemic reperfusion injury where it confers protection [83].

Several studies reported activation of the Nrf2 pathway by arsenite, which is consistent with its reactivity towards cysteines, and gene expression profiling of human bronchial epithelial cells exposed to different concentrations of the metalloid showed induction of some Nrf2 target genes [84]. Heme oxygenase induction has been reported in vivo or in vitro in several studies with various arsenical compounds, including arsenite [85].

It should be noted, however, that the results of Nrf2 activation on GSH levels depend on the experimental model used. A recent meta-analysis of 88 studies on the effect of arsenite on Nrf2 [86] has shown that while in vivo administration of the metalloid causes a depletion of cellular GSH, in vitro it increases it. This probably reflects the overall balance between the effect of GSH depletion, either directly by the metalloid or via overproduction of ROS, and the effect of GSH synthesis, which is promoted by Nrf2. This seems supported by another recent meta-analysis of 39 in vivo studies showing that, while arsenite consistently induces Nrf2 target genes, it also induces ROS generation and depletes GSH levels [87].

The mechanism by which arsenite can activate Nrf2 is complex. Mutation of specific cysteines (particularly Cys51) on KEAP1 shows that this is important in the interaction with arsenite [88], but arsenite can also directly bind cysteines of Nrf2, and these are also important in its transcriptional activation, as shown by studies with cysteine mutants [89]. Other pathways contribute to arsenite-induced Nrf2 activation. Arsenite induces p62 accumulation due to dysregulated autophagy flux [90,91,92], and accumulation of p62 then results in the sequestration of KEAP1 in the autophagosomes, impairing Nrf2 degradation [93]. On the other hand, p62 is a downstream gene of Nrf2, therefore implying a positive feedback loop [90]. In addition, arsenite induces acetylation of Nrf2 by p300/CREB (cAMP response element-binding protein), which enhances Nrf2’s binding capacity to promoter-specific DNA [94].

Many studies reported that arsenite increases Nrf2 mRNA levels [87], suggesting additional mechanisms to the classical transcriptional activation mediated by regulation of Nrf2 stability and its degradation. As will be discussed below, arsenite can promote an ER stress response and induce PERK-mediated activation of Nrf2 [95] or activating transcription factor 4 (ATF4)-dependent transcriptional regulation of Nrf2 [96].

**Figure 2:**
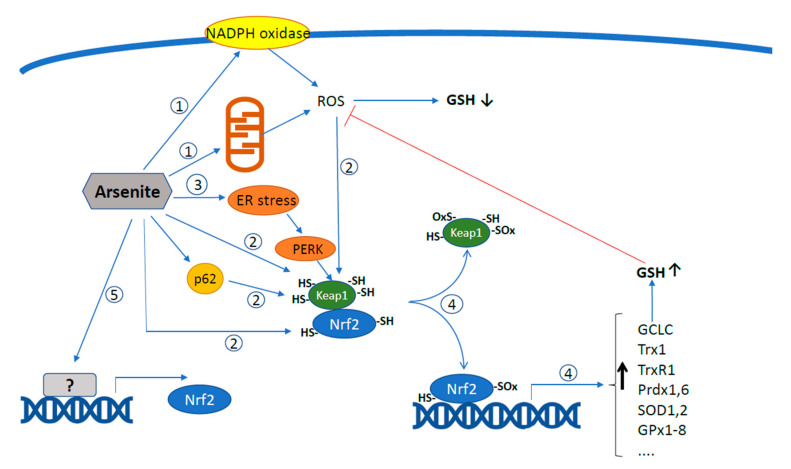
Mechanisms of Nrf2 activation by arsenite. The different pathways by which arsenite can promote the transcriptional activation of Nrf2 are highlighted. ① Arsenite stimulates ROS production by NADPH oxidase in the plasma membrane and by mitochondria; ② ROS and arsenite oxidize Keap1 and Nrf2 directly or induce Keap1 sequestration via p62; ③ arsenite causes ER stress which activates Nrf2 via PERK; ④ as a result, Nrf2 is freed, translocates to the nucleus and activates the transcription of several enzymes; ⑤ additionally, arsenite can induce neosynthesis of Nrf2 protein at the transcriptional level.

The main mechanisms by which arsenite can activate Nrf2 are highlighted in Figure 2, in which the possibility of an indirect mechanism, i.e., via the formation of ROS, is also included. A final consideration is that activation of Nrf2 is, in general, of critical importance to mitigating arsenite toxicity, although its persistent activation associated with prolonged exposure to the metalloid may, in fact, promote deleterious events enhancing its cancerogenicity [97,98].

## 5. Arsenite-Dependent Regulation of Nrf2 by Mitochondrial ROS: Impact on Survival vs. Apoptotic Signalling

It is well established that ROS can induce Nrf2 activation via a mechanism involving Keap-1 oxidation and suppression of Keap-1-dependent Nrf2 degradation [79,80]. Thus, as indicated in the previous section, arsenite can activate Nrf2 through this indirect mechanism [91,92,99] driven by both NADPH-oxidase- and mitochondria-derived ROS. Although it is at present unclear whether these two mechanisms similarly or differentially activate Nrf2, the diffusible nature of H_2_O_2_ is consistent with the possibility of an effective and similar contribution.

The notion that mitochondrial ROS activates Nrf2 is well established [100,101], and it is also clear that Nrf2 target genes can exert beneficial effects in mitochondria through different mechanisms, which include up-regulation of the mitochondrial antioxidant defence [76], mitophagy [78,102], and mitochondriogenesis [74,77,78].

These findings, therefore, imply a beneficial role of mitoO_2_^•−^, mediated by different mechanisms converging into Nrf2 activation-dependent mitohormesis [100]. Given the well-established toxic potential of mitoO_2_^•−^, it appears reasonable to predict that the protective signalling will prevail under conditions in which mitoO_2_^•−^ is generated transiently and in limited amounts. While an excess of mitoO_2_^•−^ promotes toxicity, based on both excessive damage and inhibition of Nrf2 signalling [100,101], it is likely that intermediate levels of mitoO_2_^•−^ cause both opposing responses. This is consistent with the notion that the Nrf2 signalling mediates an adaptive response aimed at preventing/mitigating, and delaying, the onset of cell death. Clearly, we might also expect the contribution of an array of variables in the regulation of this delicate equilibrium.

Our previous work using well-defined conditions, in which arsenite generates mitoO_2_^•−^, established a link between these toxic events and Nrf2 activation and the ensuing enhanced expression of target genes as γ-glutamylcysteine synthase and increased GSH levels [55].

However, as previously discussed, the same species also mediate other events, such as an early DNA strand scission [54] and the delayed induction of mitochondrial dysfunction associated with the triggering of MPT-dependent apoptosis [56]. It was, therefore, interesting to observe that these events were anticipated, with remarkably lower arsenite concentration requirements, under conditions in which the increased GSH biosynthesis was blunted.

Thus, arsenite promotes mitoO_2_^•−^ formation under conditions in which pro-survival Nrf2-dependent as well as MPT-dependent apoptotic signalling responses are sequentially generated, and the triggering of Nrf2 significantly limits and delays the ensuing apoptosis (Figure 1). A final consideration is based on the fact that, as previously discussed, the possibility that arsenite generates mitoO_2_^•−^ is heavily conditioned by the specific functional organisation of the ER in different cells [56], thereby implying a cell type dependence also for the Nrf2 response driven by these species.

## 6. Crosstalk between Arsenite-Induced ER Stress, UPR, and Nrf2-Mediated Antioxidant Responses

ER stress, triggered by the accumulation of unfolded or misfolded proteins, activates a homeostatic response, the unfolded protein response (UPR), which aims at reestablishing the ER homeostasis. As the term UPR suggests, unfolded proteins, resulting from a defect between the load of proteins to be folded into the ER and the capacity of the organelle to fold them, activate the stress in the organelle with the consequent response [103].

UPR is a multifaceted response that improves the ability of ER to fold proteins by increasing from one side its chaperoning and degradation capacity, mostly up-regulating the dedicated chaperones/enzymes, and from the other side by promoting the attenuation of the protein translation.

UPR is initiated by the following three ER membrane receptors: inositol-requiring enzyme (IRE1), protein kinase R-like endoplasmic reticulum kinase (PERK), and activation transcription factor 6 (ATF6), which regulate the three related pathways of signal transduction. The most ancient of the three is IRE, which promotes the unconventional splicing of X-box-binding protein 1 (XBP1) mRNA, resulting in the translation of this transcription factor, which finally regulates the so-called UPR target genes. ATF6 is proteolytically activated, thereby translocating to the nucleus and acting as a transcription factor for chaperones such as BIP (GRP78). PERK is an initiation factor 2 (eIF-2) kinase that promotes a general attenuation of protein translation by phosphorylating eIF-2 and the selective translation of ATF4, which is upstream to the pro-apoptotic C/EBP homologous protein CHOP and its two targets ERO1α and GADD34 [104,105].

Of note, ERO1α is a protein disulfide oxidase that, in virtue of its role in protein oxidative folding, is up-regulated during UPR. However, ERO1α generates a stoichiometric amount of H_2_O_2_ in its catalytic reaction of electron relay with PDI aimed at introducing disulfide bonds in client proteins, thereby generating a high amount of this oxidant in highly secretory cells [106]. For this purpose, an analysis with fluorescent probes, which measure ROS in the ER, suggested that this organelle potentially generates an amount of ROS even greater than that generated by mitochondria, further supporting the hypothesis that ER and ERO1α activity may represent an important source of H_2_O_2_/ROS [107].

Different proteins/enzymes into the ER were suggested to counteract the ERO1α-generated H_2_O_2_ or the effects of the oxidant on protein targets. For example, peroxiredoxin IV, which is a 2-Cys peroxiredoxin, reduces the H_2_O_2_ in water, also promoting PDI re-oxidation in the absence of ERO1 and thus participating in the disulfide bond formation of newly synthesised proteins into the ER [108]. SEPN1, a type II selenocysteine-containing membrane protein of the ER, counteracts the ERO1α-mediated hyperoxidation of the sarcoplasmic reticulum calcium pump SERCA2 in a redox-dependent manner, finally promoting the activation of this calcium pump [109,110]. Therefore, the local oxidative activity of ERO1α is counteracted by other proteins/enzymes localised into the ER.

However, ERO1α, by increasing Ca^2+^ release from the ER, could also promote Ca^2+^-dependent ROS formation in various subcellular compartments, including the mitochondria [69,111,112], thereby implying that the UPR, by activating PERK and then ERO1α, regulates the ROS-dependent Nrf2 antioxidant response.

Additionally, activated PERK can also promote the direct phosphorylation of Nrf2 and its transcriptional activation [95]. The crosstalk between PERK signalling and Nrf2-mediated antioxidant response is further supported by the evidence pointing that impaired signalling in this branch of the UPR is due to genetic mutations in both PERK and ATF4, which markedly raises the levels of ROS in ER-stressed cells [113].

The UPR signalling network, also converging in the activation of autophagy [114,115], is stimulated by ER stress to restore homeostasis and hence survival [114,115,116]. On the other hand, under conditions of sustained or prolonged ER stress, the UPR and/or autophagy promote apoptosis [115,116,117]. Many of the above pathways converge at different levels, with potentially significant impacts in different toxicity paradigms.

Substantial evidence in the literature documents the ability of arsenite to promote ER stress, eventually associated with the triggering of apoptosis [33,34,35,45,118,119]. The mechanism mediating ER stress activation is, however, poorly defined, and the specific relevance of direct effects of the metalloid remains elusive. The possibility of indirect effects mediated by ROS is also poorly understood, with very few details, if any, on the relative impact of ROS derived from different sources. Limited information is also available in the opposite direction, i.e., on the impact of ER stress on ROS formation, in particular on the mechanisms regulating Ca^2+^ homeostasis in microdomains, in which ROS formation takes place.

We have previously briefly discussed the close contact existing between the ER and the mitochondria, and the resulting crosstalk between these two organelles [56]. mitoO_2_^•−^-derived H_2_O_2_ can easily reach the ER and promote effects such as ER stress and Ca^2+^ release from either the IP_3_R, RyR, or both, as a consequence of direct oxidation of specific thiols present in both channels [120,121,122,123]. In an opposite direction, IP_3_R- and/or RyR-derived Ca^2+^ might instead be taken up by mitochondria, thereby boosting mitochondrial ROS emission [25,124,125].

Our previous work demonstrated that arsenite-induced mitoO_2_^•−^ formation causes an initial stimulation of Ca^2+^ release from the IP_3_R, critically connected with the triggering of further Ca^2+^ release from the RyR, apparently mediated by ERO1α [69]. On the other hand, ERO1α activation/expression was causally linked to Ca^2+^ mobilisation from the IP_3_R and RyR, which leads to the conclusion that ER stress and ERO1α are part of a positive amplification loop leading to Ca^2+^ mobilisation and accumulation in mitochondria to build-up mitoO_2_^•−^ formation in these organelles. It is instead still unclear whether mitochondrial ROS contributes to ERO1α activation/expression. In principle, they should, although a specific investigation to provide an answer to this question has yet not been performed.

In any case, ERO1α promotes ROS formation in situ into the ER as well as in mitochondria, possibly linked to RyR sensitization and to the ensuing mitochondrial accumulation of Ca^2+^ [69]. This crosstalk may, therefore, at the initial stages, promote survival through the activation of the Nrf2 signalling. Likewise, it can be predicted that the persistence of these events will rather lead to toxicity associated with the induction of mitochondrial dysfunction. Under these conditions, the reduced rate of ATP formation may further compromise the Ca^2+^ buffering capacity of the ER, thereby fueling the amplification loop involved in the regulation of the above crosstalk. This will eventually cause Ca^2+^ overload in the mitochondria, which in turn, is associated with the induction of MPT and the ensuing apoptotic signalling [126,127]. As a final note, we also reported that arsenite-induced ER stress is associated with autophagy and that inhibition of the autophagic process partially prevents mitochondrial dysfunction and toxicity [60].

Thus, the ER stress response induced by arsenite is critically connected with the formation of mitochondrial ROS and, hence, with the ROS-dependent activation of the Nrf2 protective signalling and ROS-dependent toxicity.

## 7. Conclusions

The general idea deriving from the analysis of numerous studies, in which the source of ROS was not always determined, is that arsenite promotes an array of effects via multiple mechanisms, which crosstalk at different levels. In particular, numerous mechanisms whereby arsenite induces Nrf2 expression interconnected at various levels with ER stress signalling responses have been thus far proposed. The overall scenario is therefore confusing, most likely as a consequence of the chemical nature of the metalloid, inducing a wide spectrum of effects through its binding to protein thiols and ROS formation.

We tried to connect the above information with our findings obtained with arsenite in well-controlled cellular systems characterised by the unique formation of mitoO_2_^•−^. The overall mechanism emerging from these studies is shown in Figure 1.

Arsenite promotes mitoO_2_^•−^ formation via a mechanism requiring interactions with the mitochondrial respiratory chain and the ER. The crosstalk between the ER and mitochondria is critically influenced by their functional and spatial organisation. Arsenite causes an initial IP_3_R activation and a successive stimulation of the RyR, critically mediated by an ERO1α-dependent mechanism, leading to the mitochondrial accumulation of Ca^2+^. MitoO_2_^•−^-derived H_2_O_2_ mediates mitochondrial dysfunction and toxicity. The early ER stress response is also critically connected through mitoO_2_^•−^ formation with the triggering of the Nrf2 cytoprotective signalling, which indeed significantly mitigates and delays the onset of MPT-dependent apoptosis.
